# Hepatotoxicity of Nonsteroidal Anti-Inflammatory Drugs: A Systematic Review of Randomized Controlled Trials

**DOI:** 10.1155/2018/5253623

**Published:** 2018-01-15

**Authors:** Pajaree Sriuttha, Buntitabhon Sirichanchuen, Unchalee Permsuwan

**Affiliations:** Faculty of Pharmacy, Chiang Mai University, Chiang Mai, Thailand

## Abstract

**Background:**

Nonsteroidal anti-inflammatory drugs (NSAIDs) are the most widely used medication in several countries, including Thailand. NSAIDs have been associated with hepatic side effects; however, the frequency of these side effects is uncertain.

**Aim of the Review:**

To systematically review published literature on randomized, controlled trials that assessed the risk of clinically significant hepatotoxicity associated with NSAIDs.

**Methods:**

Searches of bibliographic databases EMBASE, PubMed, and the Cochrane Library were conducted up to July 30, 2016, to identify randomized controlled trials of ibuprofen, naproxen, diclofenac, piroxicam, meloxicam, mefenamic acid, indomethacin, celecoxib, and etoricoxib in adults with any disease that provide information on hepatotoxicity outcomes.

**Results:**

Among the 698 studies, 18 studies met the selection criteria. However, only 8 studies regarding three NSAIDs (celecoxib, etoricoxib, and diclofenac) demonstrated clinically significant hepatotoxic evidence based on hepatotoxicity justification criteria. Of all the hepatotoxicity events found from the above-mentioned three NSAIDs, diclofenac had the highest proportion, which ranged from 0.015 to 4.3 (×10^−2^), followed by celecoxib, which ranged from 0.13 to 0.38 (×10^−2^), and etoricoxib, which ranged from 0.005 to 0.930 (×10^−2^).

**Conclusion:**

Diclofenac had higher rates of hepatotoxic evidence compared to other NSAIDs. Hepatotoxic evidence is mostly demonstrated as aminotransferase elevation, while liver-related hospitalization or discontinuation was very low.

## 1. Introduction

Nonsteroidal anti-inflammatory drugs (NSAIDs) are the most widely used medication in several countries, including Thailand, for treatment of symptoms of pain and inflammation, such as osteoarthritis (OA) and rheumatoid arthritis (RA) [1, 2]. It has been reported that 12.1% of the US population took NSAIDs at least three times per week for more than 3 months [3]. In Thailand, NSAIDs are both widely prescribed by physicians and available for purchase over-the-counter without a physician's prescription in drug stores.

Based on the data of Thai Food and Drug Administration (Thai FDA) during 1984–2016, medications used for musculoskeletal system disorders were the second-most common cause of adverse drug events (ADE), resulting in 14% of all reported ADEs. Ibuprofen and diclofenac were listed among the top 15 drugs to cause ADE [4]. While the major adverse effects of NSAIDs such as gastrointestinal mucosa injury are well known, NSAIDs have also been associated with hepatic side effects ranging from asymptomatic elevations in serum aminotransferase levels and hepatitis with jaundice to fulminant liver failure and death [5]. In 2008, lumiracoxib was withdrawn from the market in several countries, mostly due to its potential to cause severe hepatic failure [6], which is classified as one type of hepatotoxicity.

Drug-induced hepatotoxicity leads to abnormalities in liver tests or liver dysfunction. An elevation of ALT, ALP or conjugated bilirubin was confirmed as “> 2 × ULN” according to CIOMS criteria [7, 8]. At present, the thresholds and the cutoffs for ALT have been modified; the 5 × ULN has been suggested in a recent state-of-the art paper written by international experts [9].

Hepatotoxicity is more frequently discovered during postmarketing studies or, even, much later. This is due to the slightly low incidence rate of NSAIDs associated with hepatotoxicity [5, 10, 11]. The sample size of the premarketing studies designed to assess the efficacy or safety of NSAIDs might not be sufficient to provide the true incidence rate of hepatotoxicity. Although clinically apparent liver injury from NSAIDs is rare (~1–10 cases per 100,000 prescriptions) [5], NSAIDs are consumed in massive amounts worldwide; hence, despite the overall low incidence rate of NSAID-induced hepatotoxicity, their widespread use makes them an important cause of drug-induced liver injury.

In 2005, Rostom et al. [12] conducted a systematic review of randomized controlled trials (RCT) of diclofenac, naproxen, ibuprofen, celecoxib, rofecoxib, valdecoxib, and meloxicam in arthritis patients. The authors defined hepatic toxicity as aminotransferase elevations > 3 × ULN, liver-related drug discontinuation, serious hepatic adverse events, liver-related hospitalizations, and liver-related deaths. They concluded that diclofenac and rofecoxib had higher rates of aminotransferases, three times greater than ULN when compared with either placebo or other NSAIDs. However, none of these studies found high rates of serious hepatic adverse events, hospitalization, or death. These results are in concordance with the findings reported by Rubenstein and Laine [11] who evaluated the incidence and risk of serious liver-related NSAID toxicity using published literature for population-based observational studies (case-control, controlled cohort, and single cohort population-based studies). They found that the incidence rate of hepatotoxicity associated with hospital admission was in the range of 3.1–23.4/100000 patient-years. The incidence rate of hepatotoxicity associated with NSAIDs, also obtained from a retrospective study, was found to be in the range of 1.4–9/100000 patient-years [13–15].

Therefore, randomized, controlled trials that assessed the risk of clinically significant hepatotoxicity associated with NSAIDs that are commonly used in Thailand were systematically reviewed. Those NSAIDs included ibuprofen, naproxen, diclofenac, piroxicam, meloxicam, mefenamic acid, indomethacin, celecoxib, and etoricoxib.

## 2. Methods

### 2.1. Search Methods for Identification of Studies

The relevant articles were identified by searching the following databases for data up to July 30, 2016: the Cochrane Library, EMBASE, and PubMed. A comprehensive search was systematically performed, and the search was limited to the English language. The electronic search terms are summarized in supplement 1. Manual searching for relevant publications from extracted articles was also performed.

### 2.2. Study Selection

A total of 9 NSAIDs that are commonly used in Thailand were chosen. There were ibuprofen, naproxen, diclofenac, piroxicam, meloxicam, mefenamic acid, indomethacin, celecoxib, and etoricoxib. In contrast to a previous study [12] which included randomized controlled trials of at least 4 weeks, this study did not limit the study duration of the randomized controlled trial. This is because the apparent mechanism by which almost all NSAIDs cause hepatic injury is idiosyncrasy rather than intrinsic toxicity (except acetaminophen and aspirin). As a result, the time to onset of liver injury varies from within a week to several months after starting any drugs [34]. The criteria considered for drug-induced hepatic injury in this study were elevation of transaminases (alanine aminotransferase or aspartate aminotransferase) to >3 × ULN or ALP to >2 × ULN threshold as a significant elevation because it is the most commonly used initial screen for hepatic injury [35]. Therefore, randomized controlled trials of adults (age ≥18 years) with any diseases were included for data extraction if (1) the studies described at least one of the following NSAIDs: ibuprofen, naproxen, diclofenac, piroxicam, meloxicam, mefenamic acid, indomethacin, celecoxib, and etoricoxib and (2) hepatotoxicity outcomes were reported as the number of events related to at least one of the following outcomes: elevation of aspartate aminotransferase (AST) > 3 × ULN, elevation of ALT > 3 × ULN, elevation of ALT, AST or both > 3 × ULN, elevation of ALP > 2 × ULN, Hy's case (ALT > 3 × ULN and total bilirubin > 1.5 × ULN), liver-related treatment discontinuations, and liver-related hospitalization.

**Figure 1:**
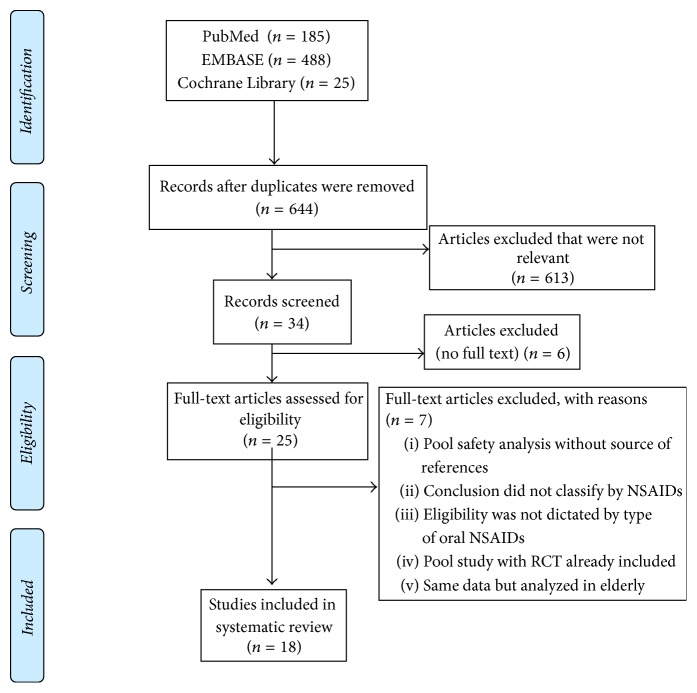
The flowchart of the selected studies in this systematic review.

Eligibility assessment was performed independently in an unblinded standardized manner by two reviewers (PS and BS) to identify potential relevant articles. Disagreement between reviewers was resolved by consensus. All duplicated studies and nonrelevant articles were excluded. Data extraction and quality assessment were then performed for all included studies (Figure 1).

### 2.3. Data Extraction and Quality Assessment

The included full text articles were reviewed, and data related to study characteristics and safety outcome were extracted. Then, all the extracted data were entered into a standardized prepared table.

The data were extracted from each of the included studies according to the following criteria: (1) characteristics of the trial participants (including gender, age, comorbidity, alcohol use, and indication of NSAIDs); (2) type of intervention (including type, dose, duration, and frequency of the NSAID); versus placebo, or versus another NSAID; and (3) type of safety outcomes that were measured, such as elevation of AST or ALT < 3 × ULN, elevation of AST or ALT > 3 × ULN, elevated total bilirubin > 2 × ULN, elevation of ALP > 2 × ULN, serum ALT elevation > 3 × ULN accompanied by a serum bilirubin elevation > 2 × ULN (Hy's case), liver-related treatment discontinuation, hospitalization due to hepatic cause and acute liver failure, transplant, or death.

To ascertain the validity of the eligible RCTs, the methodological quality of the included studies was assessed independently by two reviewers (Pajaree Sriuttha and Buntitabhon Sirichanchuen) using the Jadad score [36]. The Jadad score is composed of the following issues: (1) adequacy of randomization and concealment of allocation; (2) blinding of patients, health care providers, data collectors, and outcome assessors; and (3) extent of loss to follow-up (i.e., proportion of patients in whom the investigators were not able to ascertain outcomes). Conflicts were resolved by consensus.

### 2.4. Analysis

The characteristics of the included studies are described in detail. The hepatotoxic outcomes were identified based on the “above” criterion of this study. The percentage of hepatotoxic events was calculated and classified in terms of the study and the individual drug. The estimate of the hepatotoxic events was also pooled and displayed as a graph.

## 3. Results

Of the total of 698 studies from Medline, EMBASE, and Cochrane Library, there were 644 left after deleting duplicate studies. Of these 644 articles, 613 were discarded due to their not satisfying the study inclusion criteria, and 6 studies were deleted due to unavailability of full text.

The full text of the remaining 25 citations was retrieved and examined in more detail. It was found that 7 studies failed to satisfy the study inclusion criteria as described. Therefore, 18 studies were finally included for data analysis, as shown in Figure 1.

### 3.1. Characteristics of Included Studies

Table 1 presents the characteristics of all the included studies. Of the 18 included studies, 2 studies were presented as pooled analyses from other studies, and the remaining 16 studies were individual RCT. Four studies were placebo control trials [21, 23, 24, 29], and the remaining 14 studies were active control trials. Diclofenac was reported as having been found by 11 studies [19–25, 27, 28, 32, 33]; naproxen by 5 studies [17–19, 26, 30]; celecoxib by 4 studies [23, 28, 29, 31]; ibuprofen, piroxicam, and indomethacin by only 1 study [16, 19, 24]; and etoricoxib by 1 study [27]. In all listed NSAIDs in this study, no study of mefenamic acid was found. Two trials were presented in the form of pooled analyses from multiple studies [18, 27]. The total number of samples in the 18 studies was 45,705. The NSAID use in most of the studies were indicated in osteoarthritis (13 studies) or rheumatoid arthritis (2 studies), or both (2 studies), except for one trial with low back pain [30]. It was found that 14 trials reported a higher number of female patients than male patients, while the rest of the trials did not report the gender [18, 19, 30, 31]. The patients' ages were in the range of 18–90. A majority of the patients treated had age >50 years. All the studies provided data of use of more than 1 NSAID. The duration of intervention was 4 weeks or less for 4 studies (22.2%), 6 weeks for 1 study (5.6%), 12 weeks for 6 studies (33.3%), and 16 weeks or longer for 7 studies (38.9%). The Jadad methodological quality assessment scores ranged from 2 to 5. Two studies (11.1%) had a score of 2, and 7 studies (38.8%) had a score of 5.

### 3.2. Outcomes

All the 18 studies reported safety assessment as both clinical laboratory test results and clinical symptoms of adverse events at different time points. The biomarkers most commonly used to report were AST and ALT with 88.9% and 83.3%, respectively. Alkaline phosphatase was reported in 9 studies (50.0%) and total bilirubin was reported in 7 studies (38.9%). Two studies (11.1%) reported liver function tests without specifying whether any biochemistry markers were used. A combination of laboratory tests was used to confirm drug-induced liver injury (hepatocellular, cholestatic, or mixed type) in 9 studies (50.0%), while either AST or ALT was used to confirm the same in 6 studies (33.3%), as shown in Table 2 (see supplement 2 and Table 1 for details).

The time points for monitoring liver tests of 18 studies were mostly done at different time points from the pretreatment phases to the end of the study. In five studies, the duration of study was ranged from 2 to 12 weeks and the monitoring was done at the pretreatment phases and the end of the study [17, 23, 25, 29, 30].

It was found that a variety of criteria was used for hepatotoxicity assessment in the various included studies. Eight studies did not mention the criteria in their methodology, 2 studies indicated the criteria as “marked change from baseline,” and 5 studies used 2-3 × ULN of aminotransferase as cut points. Only 3 studies used the combination of aminotransferase elevation > 2-3 × ULN with either ALP > 1.5–2 × ULN or total bilirubin > 2 × ULN as cut points to justify and assess the abnormality of liver function.

### 3.3. Evidence of Hepatotoxicity

Clinically significant evidence of hepatotoxicity was found in 8 studies [16, 20, 23, 25, 27, 28, 31, 32], which accounted for 44.4%. It was found that almost all studies reported AST or ALT, which indicates hepatotoxicity. According to the criteria adopted in this study for hepatotoxicity, 7 of 8 studies (87.5%) demonstrated the elevation of either AST or ALT, or both enzymes, >3 × ULN during the study period [16, 20, 23, 25, 27, 31, 32] (Table 3 and supplement 3; Table 2 for full information). One study did not report the magnitude of elevated AST or ALT enzymes but reported liver-related discontinuation [28]. One study assessed liver injury using both AST and ALP elevations [16]. Diclofenac and etoricoxib showed >5 × ULN of aminotransferase elevations. In addition, 2 diclofenac studies reported Hy's cases [27, 32]. It was found that most studies used high doses of diclofenac, around 100–150 mg, except for 1 study that used a lower dose, in SoluMatrix dosage form [32]. In 3 studies, it was found that diclofenac users discontinued the drug due to liver-related injury [27, 28, 32].

In 8 studies with clinically significant hepatotoxicity (Table 3), the drug that caused hepatotoxicity in 1 study did not in our interest which was fenbufen [16]. For the remaining 7 studies, the drugs that caused hepatotoxicity were diclofenac (6 studies), celecoxib (2 studies), and etoricoxib (1 study).

**Figure 2:**
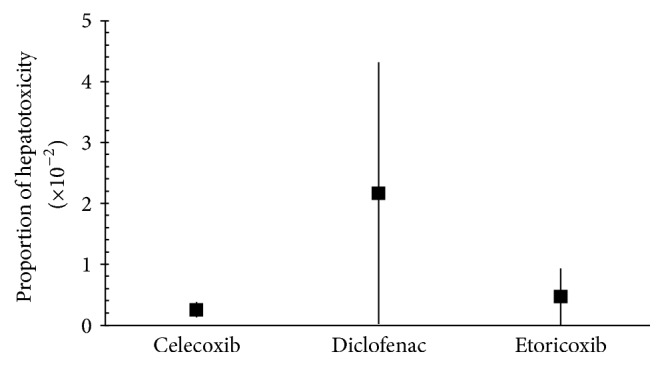
Proportion of hepatotoxicity induced by NSAIDs.

Of the total of 789 patients who received celecoxib, from 4 studies [23, 28, 29, 31], only 2 patients (0.002%) had ALT > 3 × ULN and 1 patient (0.0013%) had liver-related discontinuation. Hence, the hepatotoxicity events ranged from 0.13 to 0.38 (×10^−2^). Only 1 study reported hepatotoxicity events from etoricoxib, which were in the range of 0.005–0.930 (×10^−2^). Of those 17,412 total samples, 162 patients (0.009%) had aminotransferase elevation > 3 × ULN, 1 patient (0.00005%) had Hy's case, and 57 patients (0.0032%) had liver-related discontinuation. Compared with 2 drugs mentioned above, diclofenac had the highest proportion of hepatotoxic events which ranged from 0.015 to 4.3 (×10^−2^). Patients with AST elevation > 3 × ULN were found in 395/19998 (0.02%), ALT elevation > 3 × ULN in 864/19998 (0.04%), AST/ALT elevation > 3 × ULN in 19/19998 (0.001%), and Hy's case in 3/19998 (0.0002%), in addition to liver-related discontinuation and hospitalization in 492/19998 (0.024%) and 4/19998 (0.0002%), respectively (Table 4 and Figure 2).

## 4. Discussion

Since the clinical apparent liver injury from NSAIDs is rare and hardly found during the premarketing studies, this study was conducted to systematically review the RCTs of NSAIDs, which assessed the risk of significant hepatotoxicity. Three electronic databases (PubMed, EMBASE, and Cochrane Library) were searched from inception to July 30, 2016.

Overall 698 studies were found (185 from PubMed, 488 from EMBASE, and 25 from Cochrane Library). Only 18 studies met the selection criteria. There were several issues in those 18 included studies needed to be discussed.

The first one is the indication of NSAIDs use. Of those 18 studies, 13 studies were dispensed for the indication of osteoarthritis (OA), while 5 studies were used for rheumatoid arthritis (RA). The underlying diseases of RA and OA are different in their pathology. The inflammation of OA is distinct from that in RA. OA is chronic, comparatively low-grade, and mediated primarily by the innate immune system [37]. Therefore, the dose of NSAIDs used in OA is less than RA. As a consequence, the NSAID use in OA is less likely to influence the development of liver toxicity.

The second one is the treatment duration. We found that most studies had long-term use of NSAIDs (>12 weeks). Duration of NSAID use is not drug-related risk factor for idiosyncratic drug-induced liver injury (DILI) because this mechanism can cause DILI at any time. In relation to the occurrence of DILI, high lipophilicity and high daily dose are associated with DILI [38]. The importance is that patients should be periodically monitored appropriately.

The third one is related to the hepatotoxicity events. We found that all the hepatotoxicity events are likely to be of the hepatocellular type according to the pattern of classification of liver injuries (hepatocellular, cholestatic, and mixed hepatocellular-cholestatic) which has been defined based upon the pattern of enzyme elevations [7, 39]. This might be due to several reasons. Firstly, the number of biomarker uses considered is not sufficient. For example, the study reported only either AST or ALT; it did not report ALP. Therefore, the pattern of hepatotoxicity injury is likely to be of the hepatocellular type. However, these two biomarkers (ALT and AST) might be appropriate for evaluating diclofenac which is the most reported type of pattern of injury in the hepatocellular type. However, only two biomarkers being above (ALT and AST) is not sufficient to confirm the other two patterns of hepatotoxicity injury (cholestatic and mixed hepatocellular-cholestatic) that occur from celecoxib [40–42] or other NSAIDs such as naproxen and piroxicam [39, 43]. Assessment of liver safety data needs to take into account not only classic safety biomarkers such as the standard liver tests of ALT, AST, ALP, and total bilirubin but also patient demographics, medical history, adverse events, and concomitant medication [44]. Next, the biomarker used is not specific enough to define liver injuries. For example, the AST biomarker was used without ALT. Serum ALT is more liver-specific than AST [39]. AST can be used instead of ALT only when the latter is unavailable and when there is no known muscle pathology driving the rise in AST. In addition, time points for monitoring liver tests should be periodical since the latency of drug-induced liver injury varies. For example, the time to onset of diclofenac varies from within a week to over a year after starting the drug [39, 43, 45]. In addition, timing of the blood test is critical in defining the pattern of the enzyme elevation accurately. In some instances, an enzyme pattern that is initially hepatocellular can evolve into a mixed or even cholestatic pattern. Blood samples that are taken very early in the course of injury are more likely to show a hepatocellular pattern of injury, while samples taken late in the course of icteric cases of drug-induced liver injury are more likely to show a cholestatic pattern [39]. It was found that almost all the included 18 studies had several monitoring time points from the pretreatment phases to the end of the study.

The fourth one is related to the criteria to justify the significant hepatotoxicity events. The hepatotoxicity criteria of this study were quite rigid (elevation of ALT/AST > 3 × ULN or elevated total bilirubin > 2 × ULN, and ALP > 2 times threshold as a significant elevation; the hepatotoxic events were also defined as Hy's Law cases, liver-related treatment discontinuation, hospitalization due to hepatic cause and acute liver failure, transplant, or death). However, there was a variety of criteria used in the included studies, and these criteria might not have been rigid enough compared with this study's criteria. As a result, those studies might identify the hepatotoxicity events, but, at the same time, might not meet this study's criteria. At present, the consensus on criteria for DILI increases the cut-off level of ALT elevation to 5 × ULN. Therefore, it is more likely to exclude clinically unimportant and self-limited drug-related events as well as nonalcoholic steatohepatitis not related to DILI [9]. If these updated criteria were chosen, it would be that only two of the studies reported ALT elevation > 5 × ULN [20, 27].

The fifth one is the daily dose used for NSAIDs. The mechanism of liver injury from NSAIDs is not well understood, and it has been proposed that acidic moiety of NSAIDs or reactive adducts of NSAID metabolites may bind to host proteins and cause cellular injury in susceptible individuals [45]. Aithal and Day [46] proposed a multistep theory for diclofenac-induced liver injury; the liver injury was dose-dependent and seen mostly at the dose of 150 mg or higher. Additionally, Rostom et al. [12] found that increased doses did appear to increase the risk of elevated levels of aminotransferases with diclofenac. The results of this study support this content because a daily dose of 150 mg was reported in 8 of 11 diclofenac studies.

The findings of our study indicate that, from 18 studies, only 8 studies with 3 NSAIDs (celecoxib, etoricoxib, and diclofenac) reported clinically significant hepatotoxicity based on the hepatotoxicity justification criteria [16, 20, 23, 25, 27, 28, 31, 32]. Of all the hepatotoxicity events found from those 3 NSAIDs, diclofenac had the highest proportion which was in the range of 0.015–4.3 (×10^−2^), followed by celecoxib which was in the range of 0.13–0.38 (×10^−2^), and etoricoxib which was in the range of 0.005–0.930 (×10^−2^). On the other hand, no study was found to have reported hepatotoxicity from mefenamic acid. This might be due to infrequent use of mefenamic acid in chronic diseases.

Our findings were not in line with the systematic review conducted by Rubenstein and Laine [11] which indicated no hepatotoxicity from diclofenac. This might be due to differences in the research design (RCTs versus observational studies) and more outcome measurement in this study (elevation of aminotransferase, total bilirubin, ALP, Hy's Law cases, liver-related treatment discontinuation, hospitalization due to hepatic cause and acute liver failure, transplant, or death) than in Rubenstein's study (hospitalizations and deaths). Another systematic review of RCTs conducted by Rostom et al. [12] reported hepatotoxicity from diclofenac justified by elevation of aminotransferases (ALT/AST), which agreed with the findings of this study. However, Rostom et al. [12] did not find any hospitalization from diclofenac, which was in contrast to this study's findings.

Celecoxib and etoricoxib seem to be associated with lesser risk of liver damage even though the quality of the available data is inadequate to define accuracy of incidence [47]. Soni et al. [48] confirmed low incidence of hepatotoxicity by pooling the results of 41 RCTs. In addition, in a study conducted by Silverstein et al. [49], the RCT of celecoxib, compared with other NSAIDs, showed an increase in transaminase enzyme in 0.6% of samples, of which 0.02% had elevated ALT > 3 × ULN. These pieces of evidence were in line with the findings of this study which indicated very low proportions of clinically significant hepatotoxic events (0.0013–0.003).

As for etoricoxib, which has not been approved in the USA, 1 study reported hepatotoxicity in this systematic review. Up until present, no case of etoricoxib-induced severe hepatotoxicity has been published in PubMed. This apparent low rate of liver injury induced by etoricoxib was due to the fact that most studies analyzing this drug were underpowered to detect clinical events [47]. However, warning about potential hepatotoxicity is written in the product summary.

In summary, to report liver safety assessment from randomized controlled trials, the requirements for the studies should be uniform; for example, necessary criteria such as precise definitions and report outcome should be clearly specified. We found that, among 9 NSAIDs, diclofenac has the greatest proportion of hepatotoxic events, with low liver-related hospitalization. To minimize potential risk of hepatotoxicity from NSAIDs, especially diclofenac, the lowest effective dose is recommended and avoid dispensing those NSAIDs as the first-line drug if other safer NSAIDs are available.

## Figures and Tables

**Table 1:** Characteristics of studies included in systematic review.

Number	Study	Study design	Intervention (dose/day)	Characteristics of patients				Duration (weeks)	Jadad score
				Number of samples	Female (%)	Age (mean ± SD) (years)	Indication		
1	Buxton et al. 1978 [16]	A crossover, double-blind, two cycles of four-week treatment study	Indomethacin 75 mg	20	70.0	64	OA	2 wk fenbufen 2 wk placebo 2 wk indomethacin	3
			Fenbufen 600 mg	20					

2	Verbruggen et al. 1982 [17]	A crossover, double-blind study	Naproxen 500 mg	11	90.0	66 (median)	OA	2	3
			Nabumetone 1000 mg	10	64.0	62.5 (median)			

3	Turner 1988 [18]	Two randomized, double-blind studies	Naproxen 750 mg	286	N/A	52.3 (all)	RA	24	3
			Naproxen 1500 mg	300					

4	Eversmeyer et al. 1993 [19]	A randomized, open-label, multicenter study	Diclofenac 100–200 mg	296	N/A	N/A Age > 18	OA and RA	12	2
			Nabumetone 1500–2000 mg	3315					
			Naproxen 500–1500 mg	279					
			Ibuprofen 1200–3200	296					
			Piroxicam 10–20 mg	286					

5	Kennedy et al. 1994 [20]	A randomized, double-blind, parallel, multicenter study	Diclofenac 150 mg	121	70.0	64.6 ± 9.7	OA	16	5
			Ketoprofen ER 200 mg	118	70.0	63.3 ± 10.8			

6	Schmitt et al. 1999 [21]	A randomized, double-blind, multicenter study	Diclofenac, enteric coated 150 mg	112	98.9	60 ± 9	OA	12	5
			Diclofenac dual release capsule 150 mg	111	82.9	61 ± 9			
			Diclofenac capsule 75 mg	114	85.1	61 ± 10			
			Placebo	56	82.1	62 ± 9			

7	Morgan et al. 2001 [22]	A randomized, double-blind, parallel, multicenter study	Diclofenac 100–150 mg	168	70.0	72 ± 6	OA	12 wk	3
			Nabumetone 1000–2000 mg	167	71.0	72 ± 6			

8	McKenna et al. 2001 [23]	A placebo-controlled, randomized, double-blind comparison study	Celecoxib 200 mg	201	68.0	61.9	OA	6 wk	4
			Diclofenac 150 mg	199	62.0	62.7			
			Placebo	200	66.0	60.4			

9	Furst et al. 2002 [24]	A randomized, double-blind, double-dummy, parallel study	Diclofenac 150 mg	181	77.9	54.7 ± 12.8	RA	12 wk	4
			Meloxicam 7.5 mg	175	78.9	56.3 ± 11.5			
			Meloxicam 15 mg	184	75.5	55.6 ± 12.1			
			Meloxicam 22.5 mg	177	73.4	56.7 ± 11.8			
			Placebo	177	75.1	56.0 ± 12.1			

10	Tugwell et al. 2004 [25]	A randomized, double-blind, double-dummy, equivalence study	Diclofenac 150 mg	311	57.0	63 ± 10	OA	12 wk	5
			Topical diclofenac 1.5% w/w 1.55 ml	311	57.0	64 ± 10			

11	Temper et al. 2006 [26]	A randomized, double-blind, single-dummy, control parallel, multicenter study	Naproxen 750 mg	Group 1, 239; and group 2, 52	71.8	59.5	OA	group 1, 52 wk; and group 2, 42 wk	5
			Acetaminophen 4 g	Group 1, 237; and group 2, 53	66.6	59.1			

12	Laine et al. 2009 [27]	Three randomized, double-blind studies: the MEDAL study, EDGE study, and EDGE II study	Diclofenac 150 mg	17,289	74.2	63.2 ± 8.5	OA and RA	72	3
			Etoricoxib 60 or 90 mg	17,412	74.2	63.2 ± 8.5			

13	Dahlberg et al. 2009 [28]	A randomized, double-blind, parallel, multicenter study	Celecoxib 200 mg	463	68.0	71	OA	52	5
			Diclofenac 100 mg	462	69.0	71			

14	Sampalis and Brownell 2012 [29]	A randomized, double-blind, placebo, and active comparator controlled pilot study	Celecoxib 200 mg	15	67.0	57.6 ± 12.6	OA	12	4
			UP446 250 mg	15	67.0	62.8 ± 10.8			
			UP446 500 mg	15	60.0	54.6 ± 14.8			
			Placebo	15	67.0	55.3 ± 14.3			

15	Shell et al. 2012 [30]	A randomized, double-blind, controlled study	Naproxen 250 mg	42		18–75	Low back pain	4	3
			Medical food alone	43					
			Both medical food and naproxen	44					

16	Chopra et al. 2013 [31]	A randomized, double-blind, parallel, multicenter study	Celecoxib 200 mg	110	NA	56.6	OA	24	5
			SGCG & SCG	220		55.5			
			Glucosamine 2 g	110		55.3			

17	Altman et al. 2015 [32]	An open-label, multicenter study	Low dose SoluMatrix diclofenac 75–105 mg	601	59.7	61.9	OA	52	2

18	Pinsornsak et al. 2015 [33]	A randomized, double-blind, controlled study	Diclofenac 75 mg	33	90.3	58.2	OA	4	5
			Sahastara (SHT) 3000 mg	33	90.0	60.4			

**Table 2:** Reported biochemistry markers and evidence of hepatotoxicity.

Laboratory test for screening hepatotoxicity	Number of studies (*n* = 18)	%
*Individual laboratory test*		
AST	16	88.9
ALT	15	83.3
ALP	9	50.0
Total bilirubin	7	38.9
Reported as liver function tests	2	11.1
*Combined laboratory test*		
AST or ALT	6	33.3
AST and ALP and total bilirubin	1	5.5
AST or ALT and ALP	3	16.7
AST or ALT and total bilirubin	1	5.5
AST or ALT and ALP and total bilirubin	5	27.8
*Evidence of hepatotoxicity*		
No evidence, or not clinically significant	10	55.6
Evidence reported, with clinical significance	8	44.4

**Table 3:** Outcome of studies indicating hepatotoxicity in this systematic review.

Number	Study	Intervention (dose/day)	Number of patients included	Hepatotoxic outcome Number of events (percentage of events)						
				AST > 3 ULN	ALT > 3 ULN	ALT, AST, or both > 3 ULN	ALP > 2 ULN	Hy's cases^a^	Liver-related discontinuation	Liver-related hospitalization
1	Buxton et al. 1978 [16]	Fenbufen 600 mg	20	2 (10.0)^c^			3 (15.0)			

5	Kennedy et al. 1994 [20]	Diclofenac 150 mg	121			14 (12.0) 5 (4.1)^d^				

8	McKenna et al. 2001 [23]	Diclofenac 150 mg	199	2 (1.0)	5 (2.5)					

10	Tugwell et al. 2004 [25]	Diclofenac 150 mg	311	4 (1.4)	13 (4.7)					
		Topical diclofenac 1.5% w/w 1.55 ml	311	1 (0.4)	3 (1.1)					

12	Laine et al. 2009 [27]	Diclofenac 150 mg	17289	246 (1.4) 104 (0.6)^c^ 31 (0.2)^e^	511 (3.0) 228 (1.3)^c^ 83 (0.5)^e^			2 (0.012)	461 (2.7)	4 (0.023)
		Etoricoxib 60 mg or 90 mg	17412			116 (0.7 ) 38 (0.2)^c^ 8 (0.05)^e^		1 (0.006)	57 (0.3)	

13	Dahlberg et al. 2009 [28]	Diclofenac 100 mg	462						8 (1.7)	
		Celecoxib 200 mg	463						1 (0.2)	

16	Chopra et al. 2013 [31]	Celecoxib 200 mg	110		2 (1.9)					
		SGCG & SCG	220		10 (4.5)^b^				7 (3.2)	

17	Altman et al. 2015 [32]	Low dose SoluMatrix diclofenac 75–105 mg	601	8 (1.4)	24 (4.1)		16 (2.7)^f^	1 (0.2)	23 (3.8)	

*Remark*. ^a^Hy's cases: ALT > 3 ULN, and bilirubin ≥ 2 ULN; ^b^more than 3–6 ULN; ^c^more than 5 ULN; ^d^more than 8 ULN; ^e^more than 10 ULN; ^f^more than 1.5 ULN.

**Table 4:** Safety outcomes of NSAIDs included in this study.

Drug	Number of trials included	Total number of patients included	Number of events							Range of hepatotoxic events^b^	Range of proportion (×10^−2^)
			AST > 3 ULN	ALT > 3 ULN	ALT, AST, or both > 3 ULN	ALP > 2 ULN	Hy's Law^a^	Liver-related discontinuation	Liver-related hospitalization		
Celecoxib	4	789	—	2	—	—	—	—	1	1-2	0.13–0.38
Diclofenac	11	19998	395	864	19	—	3	492	4	3–864	0.015–4.3
Etoricoxib	1	17412	—	—	162	0	1	57	—	1–162	0.005–0.930

*Remark*. ^a^Hy's Law: ALT > 3 ULN, and bilirubin ≥ 2 ULN; ^b^calculated from the minimal event and the maximal event due to the possibility of overlapping of patients.

## Supplementary Materials

Supplemental 1 File 1: The lists of terms used for searching in the systematic review.

Supplemental 2 File 2: The biochemistry markers used to monitor hepatotoxicity in the study.

Supplemental 3 File 3: The full information of the outcome of studies indicating hepatotoxicity in the systematic review.
