# Supplementary material for: Hepatotoxicity of Nonsteroidal Anti-Inflammatory Drugs: A Systematic Review of Randomized Controlled Trials
Source: Int J Hepatol. 2018 Jan 15;2018:5253623. doi: 10.1155/2018/5253623 (PMC5820561; doi:10.1155/2018/5253623)
Supplement: Supplemental 1 — File 1: The lists of terms used for searching in the systematic review. [file 5253623.f1.pdf]

SUPPLEMENT 1 - Search Terms

1. NSAIDs
2. Non-steroidal anti-inflammatory agents
3. "anti-inflammatory agent, non-steroidal"
4. "cyclooxygenase inhibitors"
5. Or1-4
6. ibuprofen
7. naproxen
8. diclofenac
9. piroxicam
10. meloxicam
11. "mefenamic acid"
12. indomethacin
13. celecoxib
14. etoricoxib
15. or6-14
16. brufen
17. naprosyn
18. synflex
19. voltaren
20. cataflam
21. feldene
22. Mobic
23. Ponstan
24. Indocin
25. Celebrex
26. Arcoxia

27. Or16-26
28. "Randomized controlled trial"
29. clinical trial
30. "Randomised controlled trials"
31. Or28-30
32. Liver
33. Hepatic
34. Or32-33
35. 5 and 15 or 27 and 31 and 34
36. Add filter : Species: Human , Languages: Eng
