# Supplementary material for: Hepatotoxicity of Nonsteroidal Anti-Inflammatory Drugs: A Systematic Review of Randomized Controlled Trials
Source: Int J Hepatol. 2018 Jan 15;2018:5253623. doi: 10.1155/2018/5253623 (PMC5820561; doi:10.1155/2018/5253623)
Supplement: Supplemental 2 — File 2: The biochemistry markers used to monitor hepatotoxicity in the study. [file 5253623.f2.pdf]

Supplement 2

Table 1 Biochemistry markers used in the study

No	Study	Study design	AST	ALT	ALP	Bilirubin	N/A	note
1	Buxton, et al.1978	A crossover, double-blind, two 4-weekly treatment study	/		/	/		
2	Verbruggen, et al.1982	A crossover, double-blind study					/	Hepatic function
3	Turner et al. 1988	Two randomized, double-blind studies	/	/		/		
4	Eversmeyer, et al.1993	A randomized, open-label, multicenter study	/	/				
5	Kennedy, et al. 1994	A randomized, double blind, parallel, multicenter study	/	/				
6	Schmitt , et al.1999	A randomized, double-blind , multicenter study	/	/	/	/		
7	Morgan, et al.2001	A randomized, double-blind, parallel,multicenter study	/	/	/	/		
8	McKenna , et al.2001	A placebo-controlled, randomised, double-blind comparison	/	/				
9	Furst, et al.2002	A randomized, double-blind, double- dummy, parallel study	/	/	/			
10	Tugwell , et al.2004	A randomized, double-blind, double-dummy, equivalence study	/	/				
11	Temper , et al.2006	A randomized, double-blind, single-dummy, control parallel, multicenter study	/	/				
12	Laine, et al.2009	Three randomized, double-blind studies: the MEDAL study, EDGE study, and the EDGE II study	/	/	/	/		
13	Dahlberg , et al.2009	A randomized, double-blind, parallel, multicenter study	/	/				
14	Sampalis , et al.2012	A randomized, double blind, placebo and active comparator controlled pilot study					/	Liver enz
15	Shell , et al.2012	A randomized, double-blind, controlled study	/	/	/			
16	Chopra , et al.2013	A randomized, double-blind, parallel, multicenter	/	/	/	/		
17	Altman, et al.2015	A open-label, multicenter study	/	/	/	/		
18	Pinsornsak, et al.2015	A randomized, double-blind, controlled study	/	/	/			
			16	15	9	7	2	
			88.9%	83.3%	38.9%	38.9%	11.1%	
