# Supplementary material for: Hepatotoxicity of Nonsteroidal Anti-Inflammatory Drugs: A Systematic Review of Randomized Controlled Trials
Source: Int J Hepatol. 2018 Jan 15;2018:5253623. doi: 10.1155/2018/5253623 (PMC5820561; doi:10.1155/2018/5253623)
Supplement: Supplemental 3 — File 3: The full information of the outcome of studies indicating hepatotoxicity in the systematic review. [file 5253623.f3.pdf]

Supplement 3

Table 2 Outcome of studies indicating hepatotoxicity in this systematic review [FULL INFORMATION]

No.	Study	Intervention (dose/day)	No. of patients included	Hepatotoxic outcome (Percentage of event)						
				AST > 3 ULN	ALT> 3 ULN	ALT, AST or both > 3 ULN:	ALP > 2ULN	Hy's Law (ALT > 3 ULN, and bilirubin≥ 2 ULN)	Liver-related discontinuation	Liver-related hospitalization
1	Buxton, et al.1978	Fenbufen 600 mg		2/20 (10%)(> 5 ULN)			3/20 (15%)			
5	Kennedy, et al. 1994	Diclofenac150mg	20			14/121 (12%) (> 3 ULN) 5/121 (4.1%) (> 8 ULN)				
8	McKenna , et al.2001	Diclofenac 150 mg	121	2/199 (1%)	5/199 (2.5%)					
10	Tugwell , et al.2004	Diclofenac 150 mg	199	4/311 (1.4%)	13/311 (4.7%)					
		Topical diclofenac 1.5 % w/w 1.55 ml	311	1/311 (0.4%)	3/311 (1.1%)					
12	Laine, et al.2009	Diclofenac 150 mg	311	246/17,045 (1.4) 104/17,045 (0.6) (> 5 ULN) 31/17,045 (0.2) (> 10 ULN)	511/17,053 (3.0) 228/17,053 (1.3) (> 5 ULN) 83/17,053 (0.5) (> 10ULN)			2/17,289 (0.012%)	461/17,289 (2.7%)	4/17,289 (0.023%)
		Etoricoxib 60 or 90 mg	17053			116/17,217 (0.7%) 38/17,217 (0.2%) (> 5 ULN) 8/17,217 (0.05%) (> 10ULN)		1/17,412 (0.006%)	57/17,412 (0.3%)	
13	Dahlberg , et al.2009	Diclofenac 50 mg	17217							5/458 (1.1%)
16	Chopra , et al.2013	Celecoxib 200 mg	458		2/105 (1.9%)					
		SGCG & SCG	105		10/220 (4.5%) (3-6 ULN)				7/220 (3.2%) (ALT >3 ULN)	
17	Altman, et al.2015	Low dose solumatrix diclofenac 75-105 mg	220	8/585 (1.4%)	24/585 (4.1%)			1/585 (0.2%)	23/601 (3.8%) (From increase ALT, 10 (1.7%); increase AST, 6 (1%), abnormal liver function test, 5 (0.8%) and increase hepatic enz 2 (0.3%)	
